# PEGylated thrombopoietin mimetic, JNJ‑26366821 a novel prophylactic radiation countermeasure for acute radiation injury

**DOI:** 10.1038/s41598-023-42443-0

**Published:** 2023-09-14

**Authors:** Gregory P. Holmes-Hampton, Vidya P. Kumar, Shukla Biswas, Sasha Stone, Neel K. Sharma, Betre Legesse, Justin Vercellino, Chandan Guha, Gary Eichenbaum, Sanchita P. Ghosh

**Affiliations:** 1 Armed Forces Radiobiology Research Institute, Uniformed Services University of the Health Sciences, Bethesda, MD 20889 USA; 2 Department of Radiation Oncology, Albert Einstein College of Medicine, Bronx, NY 10467 USA; 3 Johnson & Johnson, Office of the Chief Medical Officer, 410 George Street, New Brunswick, NJ 08901 USA

**Keywords:** Immunology, Pharmaceutics

## Abstract

Thrombopoietin (TPO) is the primary regulator of platelet generation and a stimulator of multilineage hematopoietic recovery following exposure to total body irradiation (TBI). JNJ‑26366821, a novel PEGylated TPO mimetic peptide, stimulates platelet production without developing neutralizing antibodies or causing any adverse effects. Administration of a single dose of JNJ‑26366821 demonstrated its efficacy as a prophylactic countermeasure in various mouse strains (males CD2F1, C3H/HeN, and male and female C57BL/6J) exposed to Co-60 gamma TBI. A dose dependent survival efficacy of JNJ‑26366821 (− 24 h) was identified in male CD2F1 mice exposed to a supralethal dose of radiation. A single dose of JNJ‑26366821 administered 24, 12, or 2 h pre-radiation resulted in 100% survival from a lethal dose of TBI with a dose reduction factor of 1.36. There was significantly accelerated recovery from radiation-induced peripheral blood neutropenia and thrombocytopenia in animals pre-treated with JNJ‑26366821. The drug also increased bone marrow cellularity and megakaryocytes, accelerated multi-lineage hematopoietic recovery, and alleviated radiation-induced soluble markers of bone marrow aplasia and endothelial damage. These results indicate that JNJ‑26366821 is a promising prophylactic radiation countermeasure for hematopoietic acute radiation syndrome with a broad window for medical management in a radiological or nuclear event.

## Introduction

Threat of nuclear attack is increased under the current geopolitical climate. To this end, contingency planning and risk mitigation is of vital importance. Such events can jeopardize the health and well-being of a population and it is vital to identify compounds to mitigate such threats. Current strategies for the general population are to triage immediately following the event and administer medical countermeasures where appropriate. First responders must also be considered as these individuals would knowingly enter a site following the occurrence of such an event, often in an effort to assess, treat, and rescue those that have been exposed. Ideally, treatment for these individuals would be in a prophylactic form and would be administered prior to exposure to circumvent the harmful effects of radiation. Individuals acting in this role can be from a varied background (different ethnicities, sexes, etc.), therefore a potential prophylactic should be effective in mitigating these effects regardless of the individual.

To date there are no FDA approved prophylactic countermeasures for hematopoietic acute radiation syndrome (H-ARS). Traditionally filings for new drugs with the FDA proceed through the investigational new drug (IND) pathway; however, radiation countermeasures and prophylactics must proceed through the Animal Rule as the approval of such products in human efficacy studies are not ethical or feasible^1^. Under this guidance efficacy of the drug should be demonstrated in more than one animal species and supporting pharmacokinetic and pharmacodynamics data must be provided.

In the late 1950s the first evidence for a hormonal regulator of thrombopoiesis was discovered in experiments showing that blood from thrombocytopenic rats transfused into healthy animals induced thrombocytosis. The factor in the blood of the thrombocytopenic rats that drove platelet elevation was discovered and called thrombopoietin (TPO)^2^. Since then, work was focused on identifying and purifying TPO^3,4^. Upon discovery of the murine myeloproliferative leukemia virus and its oncogene (*v-Mpl*) and protooncogene *c-Mpl* in the late 1980’s and early 1990’s^5,6^ the ligand was cloned and found to be TPO.

Exposure to acute total body radiation induces H-ARS, which is characterized by a decline in bone marrow and blood cells leading to neutropenia and thrombocytopenia. H-ARS is accompanied by vascular hemorrhage and bacterial translocation from the gut into the liver which is a major contributor to morbidity and mortality in the first 30 days following irradiation^7,8^. TPO is a hematopoietic cytokine and the primary regulator of platelet production and plays a major role in the proliferation of hematopoietic progenitor cells^9^. TPO administration in mice reduced the duration of thrombocytopenia and neutropenia after radiation-induced myelosuppression and increased survival in lethally irradiated mice^10,11^. TPO maintains hematopoietic stem cell viability^11^, prevents apoptosis of irradiated bone marrow cells^12^, causes expansion of the stem cell population in combination with other cytokines^13^, enhances in vivo platelet and erythroid recovery following irradiation^14^, and enhances stem cell mobilization into peripheral blood^15^. These multilineage effects support the hypothesis that a TPO agonist could effectively ameliorate radiation-induced neutropenia and thrombocytopenia, and stimulate megakaryopoiesis, and protect mice from radiation-induced lethality both pre- and post-exposure to TBI.

Clinical trials of the first generation TPO, recombinant full length human TPO (rhTPO), were conducted to treat patients with thrombocytopenic disorders following hematopoietic stem cell transplantation or conventional chemotherapy^16^. This clinical trial was discontinued because of adverse events such as neutropenia and thrombocytopenia caused by the neutralizing antibody cross-reacting with the endogenous TPO in healthy volunteers or cancer patients^16^. Since the TPO receptor (TPOR), myeloproliferative leukemia virus protooncogene (cMpl-r), is the primary target to treat thrombocytopenia, thrombopoietin mimetics were developed that have no sequence homology to endogenous TPO (e.g. Romiplostim, Eltrombopag, and JNJ‑26366821). Therefore, they do not illicit production of anti-huTPO antibodies^17^. The thrombopoietin mimetic peptide, romiplostim (Nplate) and the small molecule non-peptide mimetic eltrombopag (Promacta) have been approved by the FDA for the treatment of chronic immune thrombocytopenic purpura (ITP) patients^17–20^. Recently, Nplate has also been approved for treatment of H-ARS when administered up to 24 h post-TBI exposure^21^ but not as a prophylactic countermeasure. In an effort to test thrombopoietin mimetics as radiation countermeasures, a recent study demonstrated survival efficacy of ALXN4100TPO in a mouse model, which is a TPO receptor agonist devoid of sequence homology to TPO^7^. This drug protected mice exposed to a lethal dose of radiation, increased bone marrow cellularity as well as megakaryocytic development and accelerated multi-lineage hematopoietic recovery^7^.

JNJ‑26366821 is a novel PEGylated TPO mimetic peptide^22^, composed of two identical 14-amino-acid peptide chains joined by a lysyl residue and linked at each N-terminal to a polyethylene glycol (PEG) chain. PEGylation of the molecule reduced clearance from the system and possibility of antibody formation^22^. JNJ‑26366821 was well tolerated in healthy volunteers and PEGylation of the peptide reduces clearance of the compound without a loss of potency and the pharmacokinetic profile was investigated^22^. JNJ-26366821 showed a half-life of ~ 36h in humans^22^ and 16.3 h in mice (unpublished data). Since JNJ‑26366821 was found to be safe and well tolerated in healthy human volunteers with no evidence of antibodies against JNJ‑26366821 or endogenous TPO^22^, this promising candidate is in development as a radiation/nuclear countermeasure.

The objective of the current study was to evaluate the efficacy of JNJ‑26366821 as a prophylactic countermeasure for H-ARS in mice when administered 2–24 h prior to lethal doses of TBI. Since genetic differences influence the response of individuals to ionizing radiation^23–25^, it is important to test radiation countermeasures in more than one mouse strain to assess how efficacy might vary in different populations that have genetic variations. It is also important to test the efficacy of radiation countermeasures in both sexes to understand if there may be gender differences. Therefore, efficacy of JNJ-26366821 was evaluated in multiple mouse strains and sexes with varying radiation sensitivity (C57BL/6J males and females, and C3H/HeN males)^25^. The strains C57BL/6J and C3H/HeN are the recommended strains for testing radiation countermeasures since these strains have shown divergence in many tissue responses after irradiation^23^.

## Materials and methods

### Animals

Male CD2F1 and C3H/HeN mice (8–10 weeks old) were purchased from Envigo (Indianapolis, Indiana) and male and female C57BL/6J mice (8–10 weeks old) were purchased from Jackson Laboratories (Bar Harbor, ME). Experimental animals were identified by either tail tattoo or ear tags and housed in groups of up to 5 in plastic cages in Allentown NexGen cage systems with a total floor space of 500 cm^2^. The cages utilize an external water bottle (item # 228652-5) and a dual feeder rack (item # 225663-1). The animals received Harlan Teklad Rodent Diet 8604 and acidified water (pH 2.5–3.0) ad libitum as soon as they arrived and were acclimatized for 1–2 weeks before the start of each study. The animal rooms were maintained at 21 ± 2 °C and 50 ± 10% relative humidity with 10–15 cycles of fresh air hourly and a 12:12 h light:dark cycle.

### Ethics statement

All procedures pertaining to animals were reviewed and approved by the Uniformed Services University of the Health Sciences’ Institutional Animal Care and Use Committee (IACUC) using the principles outlined in the National Research Council’s Guide for the Care and Use of Laboratory Animals and performed in accordance with relevant guidelines and regulations^26,27^. Mice were considered moribund when they showed an inability to remain upright, were cold, unresponsive or displayed decreased or labored respiration. Moribund mice were euthanized according to American Veterinary Medical Association (AVMA) guidelines. Animal studies were conducted in compliance with ARRIVE guidelines.

### Drug preparation

JNJ‑26366821, is a novel PEGylated TPO mimetic peptide developed by Janssen Research & Development, LLC. (Raritan, NJ). JNJ‑26366821 was supplied to AFRRI in a powder form and it was formulated in sterile normal saline (0.9% NaCl) before use and protected from light^22^. Either drug or its vehicle was injected subcutaneously (SC) at the nape of the neck, pre-TBI at the time indicated for each study prior to TBI.

### Total body irradiation (TBI) studies

Mice were irradiated bilaterally at an estimated dose rate of 0.6 Gy/min in the Cobalt-60 gamma-irradiation facility at the Armed Forces Radiobiology Research Institute, Bethesda, MD in the morning (8 to 9) for all studies. These animals were placed in well-ventilated plexiglass chambers made specifically for irradiating mice. An alanine/Electron Spin Resonance (ESR) dosimetry system (American Society for Testing and Material Standard E 1607) was used to measure the dose rates in the cores of acrylic phantoms (3 in. long and 1 in. in diameter) located in all empty slots of the exposure rack in the plexiglass chamber. ESR signals were measured with a calibration curve based on standard calibration dosimeters provided by the National Institute of Standard and Technology (NIST, Gaithersburg, MD). The calibration curve was verified by inter-comparison with the National Physical Laboratory (NPL) in the United Kingdom. The corrections applied to the measured dose rates in phantoms were for decay of the Co-60 source and for a small difference in mass-energy absorption coefficients for water and soft tissue at the Co-60 energy level. The radiation field was previously reported to be uniform within ± 2%^28^.

### Housing and care of animals after irradiation

After irradiation, animals were returned to their cages and monitored three to four times daily when needed. Animals that have been irradiated experience a period of peak mortality. This period, the critical period, is typically ~ 10–14 continuous days in the range of day 8 to day 25 post-TBI (varies with mouse strains depending on their radiosensitivity). Animals that were found dead during the study were documented and removed from the cage. Any sick animals were monitored closely, and their health scored in accordance with pre-defined criteria described in the approved IACUC protocol^29^. The animals which reached humane endpoints according to predetermined health score were euthanized according to American Veterinary Medical Association (AVMA) guidelines including CO_2_ inhalation followed by confirmatory cervical dislocation^29^.

### Prophylactic survival efficacy with a single dose of JNJ‑26366821 in CD2F1

The initial survival study consisted of testing one drug dose of JNJ‑26366821 (0.3 mg/kg), one route of administration (SC), and one radiation dose (LD70/30 [70% mortality over a 30 day period] = 9.25 Gy). CD2F1 male mice were weighed, animals outside ± 10% of the mean weight were excluded. There were 24 animals (5 animals per cage, 4 in the 5th cage) per treatment group for JNJ‑26366821 and its vehicle. The mice received SC administration of either JNJ‑26366821 or saline (the vehicle) at 24 h prior to TBI. After radiation exposure, the mice were monitored daily (three times a day when necessary) for 30 days and surviving animals were euthanized at the completion of the observation period. Survival data was plotted as Kaplan–Meier plots and statistical significance of the survival differences was determined by Log-rank test using GraphPad Prism 7 software.

### Dose and time optimization, dose reduction factor of JNJ‑26366821 in CD2F1 male mice

Five doses of JNJ‑26366821 (0.1, 0.3, 1.0, 2.0, 3.0 mg/kg) were selected to determine the optimum dose of single administration of JNJ‑26366821 to achieve maximum efficacy at 24 h pre-TBI in CD2F1 mice. Mice (n = 24/group) were administered SC either JNJ‑26366821 (0.1, 0.3, 1.0, and 3.0 mg/kg) or saline 24 h before exposure at 9.75 Gy (~ LD90/30). To determine the optimum prophylactic dose of JNJ‑26366821 to achieve maximum efficacy, these doses of JNJ‑26366821 were tested at two supralethal doses (10.5 and 11 Gy) of gamma-radiation as stated above. Animals were monitored for 30-days in the same way as described previously for the survival studies.

A time optimization study was performed by selecting different time points (2, 12 and 24 h pre-TBI). Four groups including saline and three JNJ‑26366821 (0.3 mg/kg) treatment groups were used in this study. Each group (N = 24/group) was administered either JNJ‑26366821 at the specific time points or saline (only 12 h pre-TBI) before exposure to 9.75 Gy (~ LD90/30). Time response study was also performed at a supralethal dose of 11 Gy at all three time points. Animals were monitored for 30-days as described above.

To determine the dose reduction factor (DRF), male CD2F1 mice were distributed into one of twelve groups with 5 groups administered saline and 5 groups administered 1.0 mg/kg JNJ-26366821 24 h before TBI. The groups that received control article (saline) were irradiated at the following doses: 8, 8.5, 9, 9.5, and 10.0 Gy. The groups that received the test article JNJ-26366821 were irradiated at the following doses: 12, 12.5, 13, 13.5 and 14 Gy. There were 24 animals per treatment group for JNJ-26366821 and its vehicle. Animals were monitored as mentioned above. The primary endpoint of the study was 30-day animal survival. At the conclusion of the 30 days, a probit analysis (IBM SPSS Statistics 25.0) for both the control article and test article groups was performed.

### Validation of JNJ‑26366821 efficacy in C57BL/6 (males and females) and C3H/HeN (male) mice

To validate prophylactic efficacy of JNJ‑26366821 (3.0 mg/kg) in C57BL/6J mouse strains (both males and females) and C3H/HeN (males), it was administered 24 h before exposure to 8.75 Gy. There were 24 animals per treatment group for JNJ‑26366821 and its vehicle. The mice were monitored daily for 30 days and euthanized if reached humane end point according the predetermine health score described above.

### Hematological recovery with JNJ‑26366821

To study the effects of prophylactic administration of JNJ‑26366821 on the recovery from hematopoietic injury following TBI, mice (n = 10 per group) were treated with either a single dose of JNJ‑26366821 (0.3 mg/kg) or its vehicle (saline) 24 h pre-TBI at a non-lethal dose of 7.0 Gy. This dose allows the animals to recover completely without intervention from the radiation-induced hematopoietic injury. In addition, a group of sham irradiated mice were given either the drug or saline. Blood collection was performed using a 23 G needle from the submandibular vein after anesthetizing the mice with isoflurane (Hospira Inc., Lake Forest, IL) at 2 h and on days 1, 3, 7, 10, 14, 21, and 30 post-TBI. All mice were allowed to recover fully from anesthesia and monitored closely for any signs of a post-anesthesia reaction or bleeding at the collection site before returned to group housed cages. Approximately 20 µL of blood was collected in EDTA tubes and was continually rotated until CBC/differential analysis was completed using a HESKA Element HT™ 5 Analyzer system (HESKA Corporation, Loveland, CO). This CBC/differential analysis included white blood cells (WBC) counts, absolute neutrophil counts^22^, monocytes (MON), lymphocytes (LYM), red blood cells (RBC), hematocrits (HCT), and platelets (PLT) counts.

### Harvesting blood and tissues for various molecular assays

JNJ‑26366821 (0.3 mg/kg) or saline (n = 6 per group) were administered SC 24 h prior to irradiation. The experimental animals received either 0, 7 Gy (non-lethal), and 9.35 Gy (~ 70% lethal) radiation exposure at a dose rate of ~ 0.6 Gy min^−1^ in the AFRRI Co-60 gamma radiation facility. Blood was collected from the inferior vena cava under anesthesia on days 0 (2 h post-TBI), 1, 3, 7, 15, and 30 after exposure or from non-irradiated mice (at the same time points) followed by euthanasia. After which femurs and sterna were collected and processed as described below.

### Hematopoietic progenitor clonogenic assay

Clonogenicity of mouse bone marrow cells was quantified in standard semisolid cultures using 1 mL of Methocult GF + system for mouse cells (Stem Cell Technologies Inc., Vancouver, BC) according to the manufacturer’s instructions. Briefly, colony forming units (CFU) were assayed on days 0 (2 h post-TBI), 1, 3, 7, 15, and 30 after 7 Gy exposure or non-irradiated mice. Cells from three femurs from different animals were pooled, washed twice with IMDM and seeded at 1 to 5 × 10^4^ cells per 35-cm^2^ cell culture dishes (BD Biosciences). Each sample was plated in duplicate to be scored 14 days after plating. Granulocyte–macrophage colony forming units (CFU-GM), granulocyte-erythrocyte-monocyte-macrophage CFU (CFU-GEMM), colony-forming unit-erythroid (CFU-E) and erythroid burst-forming units (BFU-E) were identified and quantified following the manufacturer’s instructions. Colonies were counted 14 days after plating using a Nikon TS100F microscope. Fifty or more cells were considered one colony. Data are expressed as mean ± standard error of mean (SEM). Statistical significance was determined between irradiated vehicle treated and JNJ‑26366821-treated groups.

### Sternal histopathology

Following blood collection, animals were euthanized, and the sterna were collected on 0 (2 h post-TBI), 1, 3, 7, 15 and 30 days post-TBI. The sterna were fixed in a 20:1 volume of fixative (10% buffered formalin) to tissue for at least 24 h and up to 7 days. Fixed sterna were decalcified for 3 h in 12–18% sodium EDTA (pH 7.4–7.5) and specimens dehydrated using graded ethanol concentrations and embedded in paraffin. Longitudinal 5 μm sections were stained with regular hematoxylin and eosin (H&E) stain. A board-certified veterinary pathologist conducted blinded histopathological evaluation of these samples. The bone marrow was evaluated in situ across multiple sternebrae and graded for total cellularity and megakaryocyte numbers averaged per 10 high power fields at 40× magnification using a BX41 Olympus microscope (Minneapolis, MN). The grade scale used for cellularity is as follows: Grade 1: < 10%; Grade 2: 11–30%; Grade 3: 31–60%; Grade 4: 61–89%; Grade 5: > 90%^27^. Images were captured with an Olympus DP70 camera and imported into Adobe Photoshop (version CS5) for analysis.

### Circulating levels of EPO, TPO, and Flt3L

Serum from both 7 and 9.5 Gy (treated and untreated) was evaluated for circulatory markers of radiation-induced bone marrow aplasia. Mouse Thrombopoietin (TPO) Quantikine ELISA, mouse Erythropoietin (EPO) Quantikine ELISA and mouse/Rat Flt3 ligand (Flt3L) quantikine ELISA kits were purchased from R&D Systems Inc (Minneapolis, MN). The cytokine detection limits were 18 pg/mL^-1^, > 20 pg/mL^-1^ and > 5 pg/mL^-1^ for TPO, EPO and Flt3L ELISAs, respectively. The quantitative levels of EPO, TPO, and Flt3L were evaluated from serum samples collected on days 0, 1, 7 and 15 post-TBI following standard protocols from the vendor. Data represented are mean ± standard error of the mean for n = 6 mice^30^.

### Soluble markers of endothelial function

Serum levels of the endothelial cell adhesion molecules VCAM-1, P-selectin and E-selectin, and the gelatinase MMP-9 are commonly used to assess changes in endothelial function. Circulatory P-selectin and E-selectin, and the gelatinase MMP-9 were measured by ELISA (R&D Systems Inc., Minneapolis, MN, USA) following manufacturer’s protocol. Data presented are mean ± SEM for n = 6 mice.

### Statistical analysis

Survival data was plotted as Kaplan–Meier plots. Survival experiments utilized n = 24 animals per group. For the survival data, Fisher’s exact test was used to compare survival at 30 days and a log-rank test was used to compare survival curves with GraphPad Prism 7 software, a p value less than 0.05 was considered significant. Means and standard errors were reported for all other data. Analysis of variance (ANOVA) was used to determine if there was a significant difference among different groups. A significance level was set at 5% for each test. IBM SPSS Statistics 22 software was used for probit analysis.

## Results

### Survival efficacy of JNJ‑26366821 administered 24 h prior to total body irradiation in CD2F1 male mice, dose optimization study

**Figure 1:**
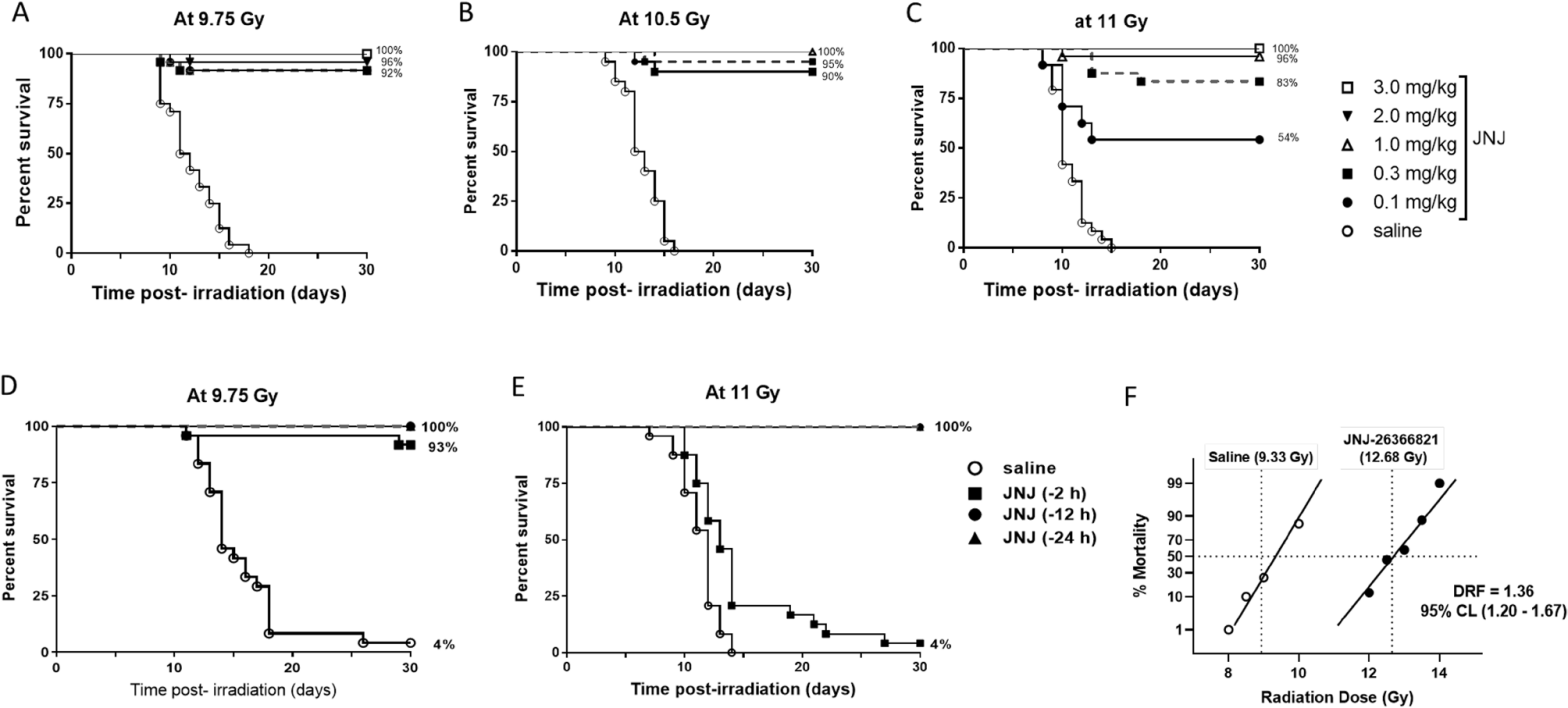
Optimization of JNJ‑26366821 dose to improve survival of lethally irradiated CD2F1 male mice and determination of dose reduction factor (DRF). Effect of increasing JNJ‑26366821 dose administered SC 24 h prior to (**A**) 9.75 Gy TBI, (**B**)10.5 Gy TBI, and (**C**) 11.0 Gy TBI (**C**) (n = 24/group). Survival curves shown here are saline as the vehicle (open circle) and JNJ‑26366821 doses 0.1 mg/kg (filled circle), 0.3 mg kg^−1^ (filled square), 1.0 mg/kg (open triangle), 2.0 mg/kg (filled inverted triangle) and 3.0 mg/kg (open square). Response of time of administration of JNJ‑26366821 to improve survival of lethally irradiated mice. Effect of JNJ‑26366821 administration SC at 2, 12 and 24 h prior to (**D**) 9.75 and (**E**) 11 Gy TBI. Survival curves shown here are saline (12 h pre-TBI) as the vehicle (open circle) and JNJ‑26366821 at 2 h (filled square), 12 h (filled circle) and 24 h (filled triangle) pre-TBI. Percent survival values on day 30 post-TBI are shown at the end of each curve. n = 24/group. Determination of DRF of JNJ‑26366821 (1.0 mg/kg) 24 h prior to exposure (**F**). A range of 6 different radiation doses were used per group. DRF was calculated from the ratio of LD50/30 values derived from probit analyses using IBM-SPSS software. Solid lines are the estimated % mortality at various radiation doses. A DRF of 1.36 was obtained for JNJ‑26366821 (1.0 mg/kg) administered 24 h prior to TBI.

To investigate the effects of JNJ‑26366821 administered 24 h prior to total body irradiation (pre-TBI), CD2F1 male mice (24 mice/group) were treated with 0.3 mg/kg JNJ‑26366821 subcutaneously and irradiated with 9.35 Gy (~ LD70/30 dose) at an estimated dose rate of 0.6 Gy/min. The study resulted in 42% survival of the saline treated group compared to 83% survival in the JNJ‑26366821 (0.3 mg/kg) treated group (Log-rank test p = 0.0061) (Supp Fig. 1). Dose response of JNJ‑26366821 on survival was studied at a range of doses (0.1–3 mg/kg) at three radiation doses (9.75, 10.5, and 11 Gy at an estimated dose rate of 0.6 Gy). We reported earlier that a single dose of 3.0 mg/kg JNJ‑26366821 was found to be safe in CD2F1 male mice in a 14-day acute toxicity study^29^. At 9.75 Gy, all mice from the saline group died by day 18 post-TBI and 92–100% survival was observed for the JNJ‑26366821 treated groups at all doses (Fig. 1A). There was no dose dependent separation of curves in this study.

To determine the optimum dose of JNJ‑26366821 for maximum survival benefit, the radiation dose was escalated to 10.5 Gy in the following study. At 10.5 Gy, no significant difference in survival among the range of JNJ‑26366821 doses (0.1–3 mg/kg) was observed, whereas all the mice in saline group died by day 16 post-TBI (Fig. 1B). Thus, a supralethal radiation dose of 11 Gy was used in this study. Total body irradiation of 11 Gy resulted in 54%, 83%, 96% and 100% survival at 0.1, 0.3, 1 and 3 mg/kg JNJ‑26366821 respectively, whereas all mice from the saline (vehicle) group died by day 15 post-TBI (Fig. 1C). From this study, highest survival efficacy was found with 3 mg/kg dose of JNJ‑26366821 with no statistically significant difference with 1 mg/kg dose (Fig. 1C).

### Survival efficacy of a single dose of JNJ‑26366821 administered pre-TBI at various times in male CD2F1 mice at 9.75 Gy—a time optimization study

To evaluate the impact of time of administration of JNJ‑26366821 (0.3 mg/kg SC) relative to TBI on survival animals (n = 24) were dosed at 2, 12 or 24 h prior to TBI 9.75 Gy (LD90/30) at an estimated dose rate of 0.6 Gy/min. JNJ‑26366821 treated groups showed almost complete survival (93–100%) in comparison with 4% survival in saline group (Fig. 1D) at 9.75 Gy. No significant difference was found between the times of administration tested. Survival efficacy of JNJ‑26366821 is 100% or almost 100% when administered in the time range of 2–24 h prior to radiation exposure. To determine the optimum time of administration of JNJ‑26366821 for maximum efficacy, the radiation dose was escalated to 11 Gy and efficacy was tested at all three times (a single dose 2–24 h prior to TBI). JNJ‑26366821 (1.0 mg/kg) was still able to rescue 100% of animals when administered at either 12 or 24 h prior to irradiation while limited (4%) survival was observed when JNJ‑26366821 was administered 2 h pre-TBI (Fig. 1E). This indicates that JNJ‑26366821 is equally effective in rescuing animals from radiation-induced mortality at the 11 Gy TBI dose when administered at either 12 or 24 h pre-TBI.

### A significantly higher DRF of JNJ-26366821 in CD2F1 male mice

Male CD2F1 mice were administered saline or JNJ-26366821 (1.0 mg/kg) 24 h prior to TBI at various radiation doses to determine the dose reduction factor (Fig. 1F). By plotting survival for the various radiation dose groups treated with either saline or JNJ-26366821, the dose of radiation correlating to 50% lethality over 30 days (LD50/30) was determined by probit analysis. For animals administered saline, the LD50/30 dose was 9.33 Gy (95% confidence interval: 9.12–9.57 Gy) and for animals administered JNJ-26366821 the LD50/30 dose was 12.68 Gy (95% confidence interval: 12.47–12.88 Gy). The DRF was calculated as 1.36 (95% confidence interval: 1.20–1.67).

### Survival efficacy of JNJ‑26366821 in C57BL/6 and C3H/HeN mice

**Figure 2:**
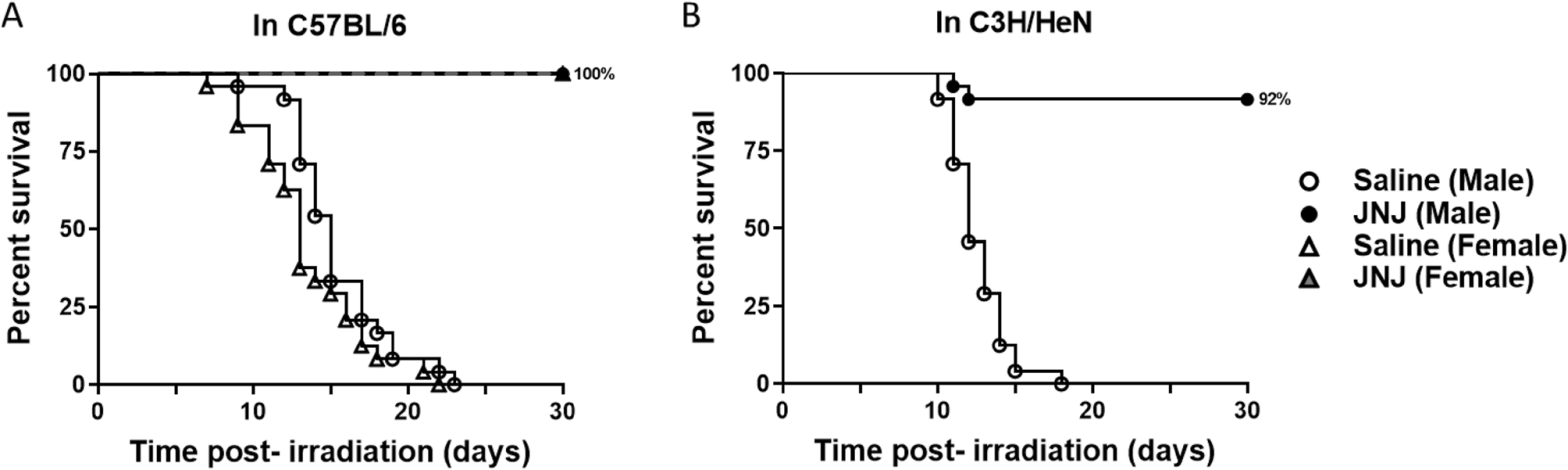
Validation of survival efficacy in multiple mouse strains and sexes. (**A**) Survival of C57BL/6J male and female mice (n = 24/group) following total body irradiation (TBI) with 8.75 Gy at the estimated rate of 0.6 Gy/min. A single dose of JNJ‑26366821 at 3 mg/kg (filled circle males, filled triangle females) or saline as a vehicle (open circle males, open triangle females) was administered 24 h prior to TBI. (**B**) Survival of C3H/HeN male mice following TBI with 8.75 Gy at the estimated rate of 0.6 Gy/min. A single dose of JNJ‑26366821 at 3 mg/kg (filled circle) or saline as a vehicle (open circle) was administered 24 h prior to TBI. n = 24/group.

In this study, efficacy of JNJ‑26366821 was tested in two other mouse strains, C57BL/6J and C3H/HeN, with differential radiation sensitivity compared to CD2F1. JNJ‑26366821 was administered (3 mg/kg) to C57BL/6J male (n = 24) and female (n = 24) mice 24 h prior to irradiation at 8.75 Gy (LD100/30) (Fig. 2A). All animals in saline treated groups (males and females) died whereas there was no mortality in JNJ‑26366821 treated groups 30 days post-TBI. The survival efficacy of JNJ‑26366821 (3 mg/kg) in C3H/HeN male (n = 24) mice was tested in mice exposed to 8.75 Gy (LD100/30) (Fig. 2B). All animals in saline treated groups died whereas there was 92% survival observed in the JNJ‑26366821 (24h pre-TBI) treated group 30 days post-TBI.

### Accelerated recovery from radiation-induced pancytopenia with JNJ‑26366821 in CD2F1 male mice

**Figure 3:**
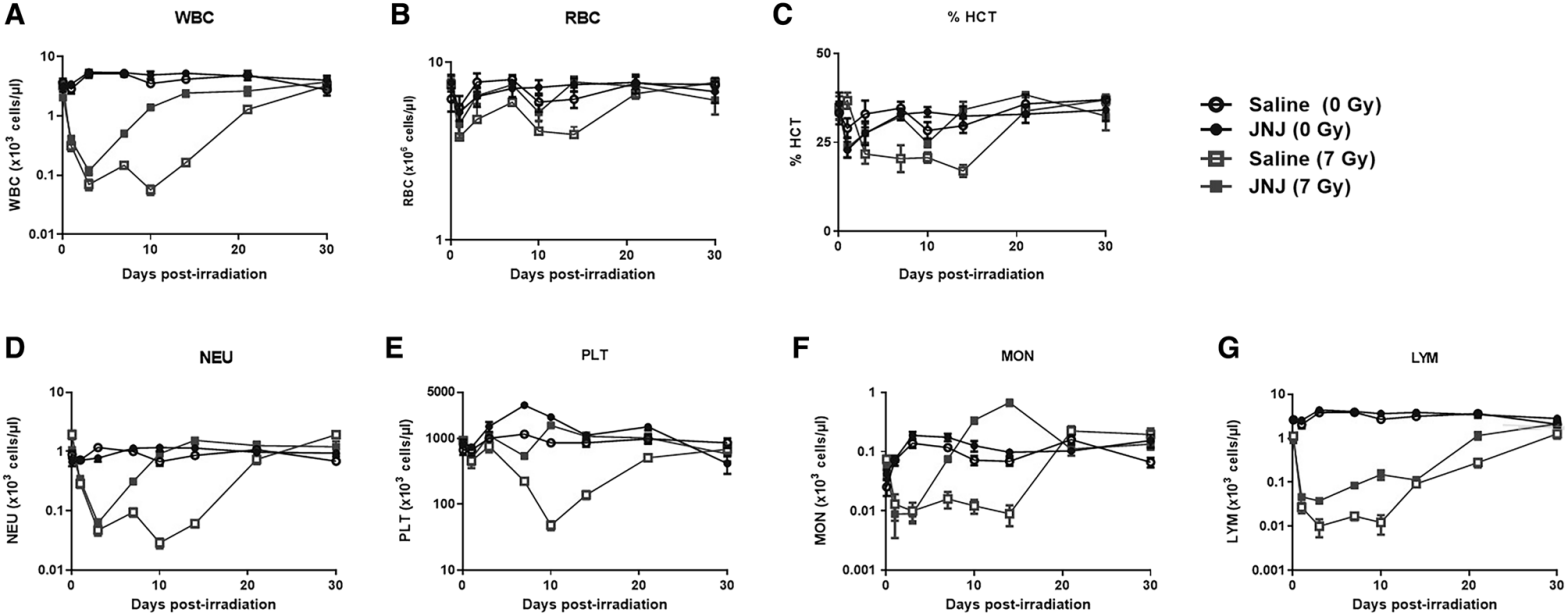
Recovery of peripheral blood cells (WBCs, RBCs, % hematocrit (%HCT), neutrophils (NEU), platelets (PLT), monocytes (MON) and lymphocytes (LYM)). Non-irradiated mice treated with saline (open circle) and JNJ‑26366821 (filled circle) and irradiated (7 Gy) mice treated with saline (open square) and JNJ‑26366821 (filled square). Either saline or JNJ‑26366821 at 0.3 mg/kg was administered 24 h prior to irradiation. Day 0 represents day of irradiation. Blood was collected on days 0 (2 h after exposure), 1, 3, 7, 10, 14, 21, and 30 post-TBI. Data represented are mean ± standard error of the mean (SEM) for n = 10 mice. Significant difference (p < 0.001–0.0125) between JNJ‑26366821 treated and saline treated irradiated groups by ANOVA is indicated with an asterisk (*). Some data points in the figure do not have error bars that are visible because they are smaller than symbols.

Peripheral blood cell recovery was studied by measuring blood cell counts, white blood cells (WBC), red blood cells (RBC), % Hematocrit (%HCT), neutrophils, platelets (PLT), monocytes (MON) and lymphocytes (LYM) of non-irradiated groups and comparing them to that of irradiated groups either treated with saline (vehicle control) or JNJ‑26366821 (Fig. 3). In the irradiated groups, by day 3 post-TBI, decline in blood cell counts were observed in both saline and JNJ‑26366821 treated groups. The recovery from pancytopenia of all blood cell counts (Fig. 3) were significant in the JNJ‑26366821 treated group, when compared to the vehicle control group. In the non-irradiated groups, significant (p < 0.05) increase in PLT was observed in JNJ‑26366821 treated groups compared to saline on days 7 and 10 post-TBI (days 8 and 11 after JNJ‑26366821 administration).

#### WBC

The WBC counts (Fig. 3A) decreased sharply reaching a nadir (0.056 × 10^3^ ± 0.009 × 10^3^ cells/μL) in the irradiated saline control group on day 10 post-TBI. On days 7, 10 and 14 post-TBI, the JNJ‑26366821 treated group showed significant recovery (day 7: 0.5 ± 0.037 cells/μL; day 10: 1.39 ± 0.1756 cells/μL; and day 14: 2.43 ± 0.32 cells/μL) as compared to the vehicle treated group (day 7: 0.15 × 10^3^ ± 0.0189 × 10^3^ cells/μL; day 10: 0.056 × 10^3^ ± 0.0097 × 10^3^ cells/μL; and day 14: 0.16 × 10^3^ ± 0.0174 × 10^3^ cells/μL). WBC counts in the irradiated vehicle-treated group remained low until day 10 post-TBI, with a slow recovery profile, whereas the cells in the JNJ‑26366821 treated group recovered faster. By day 30, all four groups had similar WBC cell counts, as the irradiation dose was non-lethal.

#### RBC and %HCT

Changes in the RBC count and % hematocrit (%HCT) in the different groups are shown in Fig. 3B and C. On day 14 the %HCT of the irradiated vehicle-treated group was significantly lower than that of the control group or irradiated JNJ‑26366821 treated group (p < 0.001). The same effect was also observed in the RBC counts, suggesting accelerated recovery of peripheral hematopoietic cells with JNJ‑26366821 treatment in irradiated mice.

#### NEU

The NEU counts (Fig. 3D) decreased sharply reaching a neutropenia nadir (0.029 × 10^3^ ± 0.0051 × 10^3^ cells/μL) in the irradiated saline control group on day 10 post-TBI. On days 7, 10 and 14 post-TBI, the JNJ‑26366821 treated group showed significant recovery from neutropenia (day 7: 0.31 ± 0.026 cells/μL; day 10: 0.92 ± 0.0721 cells/μL; and day 14: 1.54 ± 0.21 cells/μL) as compared to the vehicle treated group (day 7: 0.095 × 10^3^ ± 0.0147 × 10^3^ cells/μL; day 10: 0.029 × 10^3^ ± 0.0051 × 10^3^ cells/μL; and day 14: 0.06 × 10^3^ ± 0.0079 × 10^3^ cells/μL). NEU counts in the irradiated vehicle-treated group remained low until day 10 post-TBI, with a slow recovery profile; whereas the cells in the JNJ‑26366821 treated group recovered faster. By day 30, all four groups had similar NEU cell counts with complete recovery.

#### PLT

The platelet nadir (Fig. 3E) was reached for the irradiated vehicle-treated group on day 10 (48 × 10^3^ ± 7.12 × 10^3^ cells/μL), but the JNJ‑26366821 treated group had a significantly higher (p < 0.0001) cell count (1565 × 10^3^ ± 148 × 10^3^ cells/μL), protecting the mice from thrombocytopenia. By day 14, there was no difference between the non-irradiated JNJ‑26366821 and the irradiated JNJ‑26366821 treated group (1070 × 10^3^ ± 156 × 10^3^ cells/μL). PLT cell numbers in non-irradiated groups were found to be significantly different based on the treatment they received (either saline or JNJ‑26366821). Significantly higher PLT induction after JNJ‑26366821 treatment in the control group could be one of the possible mechanisms leading to increased survival of the animals.

#### MON and LYM

The irradiated group receiving JNJ‑26366821 showed markedly higher MON counts (Fig. 3F) than the irradiated vehicle-treated group on days 7, 10 and 14 post-TBI, and the differences were statistically significant (p < 0.001). These results indicated that the administration of JNJ‑26366821 improved peripheral blood monocyte counts in irradiated mice. There were significant (p < 0.05) increases in LYM counts (Fig. 3G) as well when mice were treated with JNJ‑26366821 compared to the vehicle-treated group on day 10 post-TBI.

### A single dose of JNJ‑26366821 protects hematopoietic progenitor cells from radiation injury when administered 24 h pre-TBI

**Figure 4:**
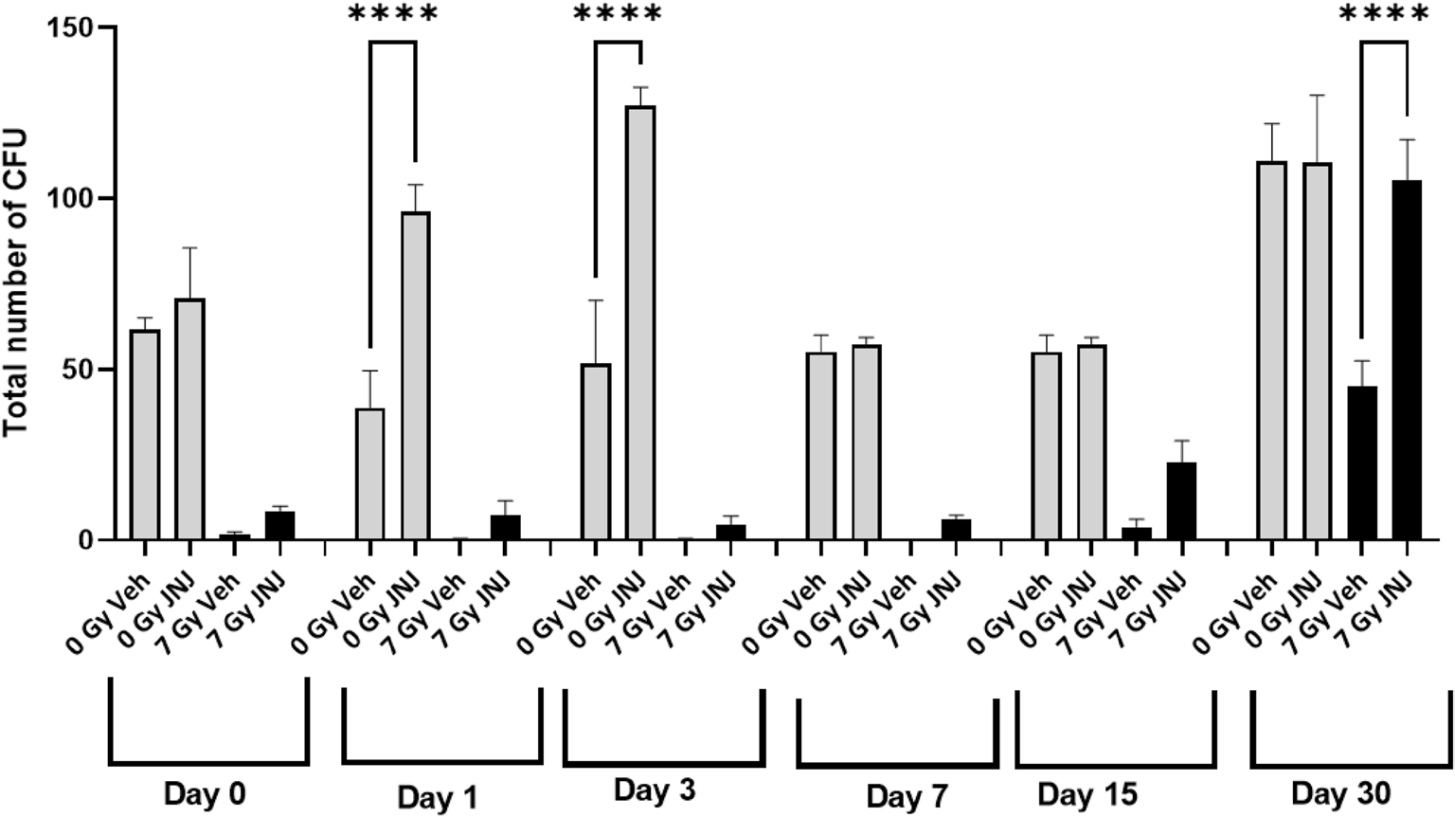
JNJ‑26366821 (0.3 mg/kg) accelerates hematopoietic progenitor cell recovery after a non-lethal dose of radiation (7 Gy) in CD2F1 mice (n = 6/group) when administered 24 h prior to total body irradiation (TBI). Clonogenic potential of bone marrow cells was assessed by a Colony forming units (CFU) assay. Femoral bone marrow was assayed on days 0 (2 h post-TBI), 1, 3, 7, 15, and 30 after exposure. Cells from three femurs were pooled, counted, and each sample plated in duplicate to be scored 14 days after plating. Data are expressed as mean ± Standard error of mean (SEM) for the total number of CFUs (CFU-GEMM + CFU-GM + BFU-E) Statistical significance (*) was determined between saline treated and JNJ‑26366821 treated groups.

In addition to detrimental effects on peripheral blood cells, irradiation also negatively affects the hematopoietic progenitor cells in bone marrow. Clonogenic assays were carried out to evaluate the extent of damage caused by irradiation and possible recovery by JNJ‑26366821 treatment administered 24 h pre-TBI. Colony forming unit (CFU) assays measured CFU-GM, CFU-GEMM, and BFU-E to evaluate the function of hematopoietic cells. CFU-E was not counted in any of the samples in this study. There was a significantly higher number of total colonies (CFU-GEMM, CFU-GM and BFU-E) were counted in the non-irradiated drug compared to saline control. Prior to day 15 post-TBI at 7 Gy, no colonies were observed in the irradiated saline treated group (Fig. 4). On day 15, the total number of colonies found in the vehicle-treated group was lower (non-significant) compared to the JNJ‑26366821 treated group. However, on day 30, total colonies were significantly lower in the vehicle compared to the JNJ-26366821 (p < 0.0001) (Fig. 4). Individual colonies were represented in supplementary Fig. 2. These data suggest that the radiation-induced depletion of hematopoietic progenitor cells can be restored by JNJ‑26366821 treatment.

### JNJ‑26366821 restores bone-marrow cellularity when administered 24 h pre-TBI

**Figure 5:**
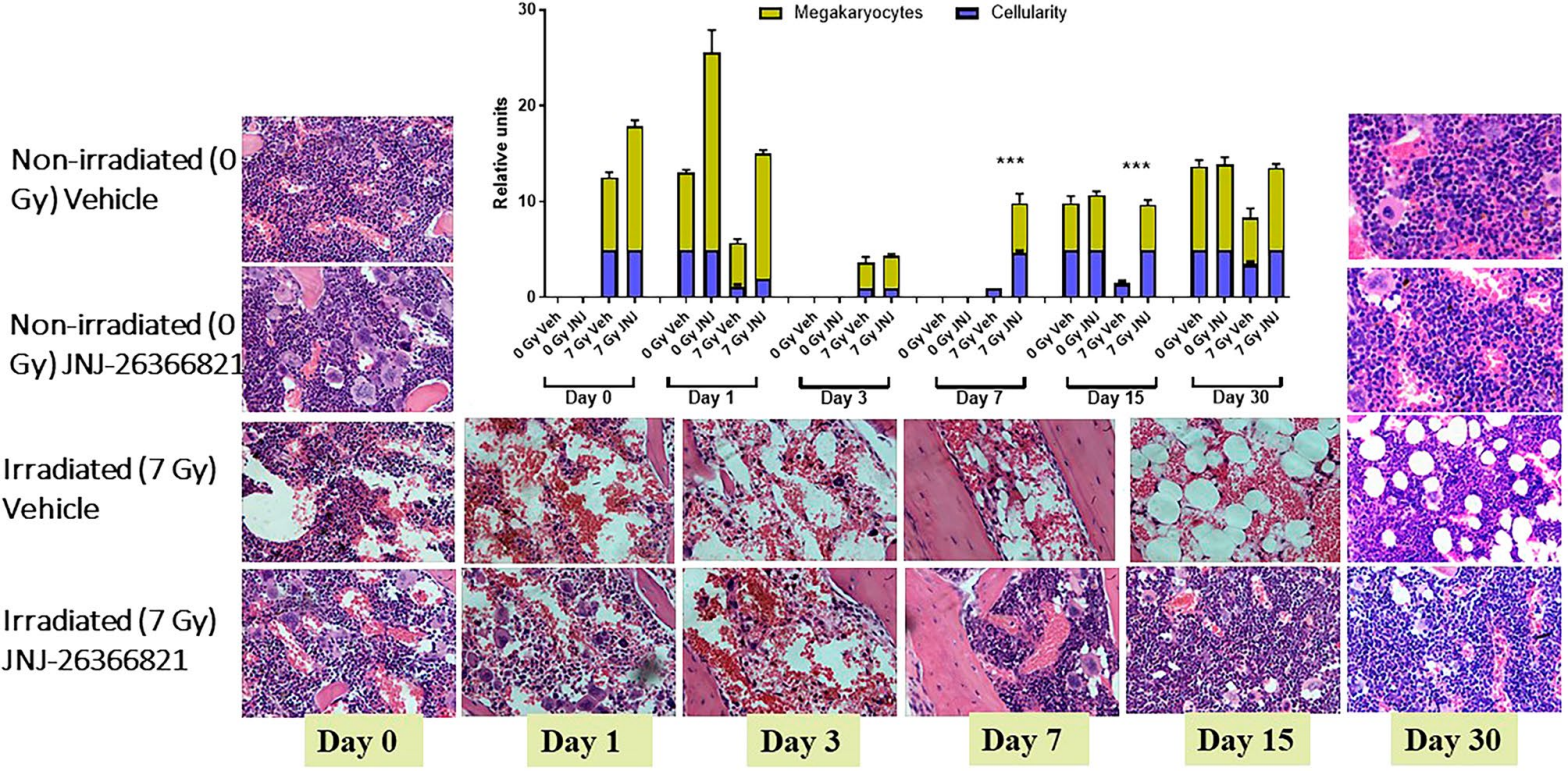
JNJ‑26366821 (0.3 mg/kg) treatment promoted sternal bone marrow hematopoietic cell recovery after non-lethal dose of total body irradiation (TBI) at 7 Gy when administered 24 h prior to TBI. Representative sternal bone marrow sections are shown for non-irradiated (0 Gy) vehicle (NRV) and JNJ‑26366821 (NRD) treated mice from days 0 and 30, and from saline (RV) and JNJ‑26366821 (RD) treated irradiated mice from days 0 (2 h post-TBI), 1, 3, 7, 15 and 30 post-TBI. Bone marrow cellularity and megakaryocyte numbers were quantitated from histological sections from days 0, 1, 3, 7, 15 and 30. Significant increase in bone marrow cellularity and megakaryocytes were observed after 7 days post-TBI in the JNJ‑26366821 treatment group. Even after 15 days post-TBI, there was significant difference in cellularity of saline treated and JNJ‑26366821 treated groups, later showing recovery from radiation injury. Data represented are mean ± standard error of the mean (SEM) for n = 6 mice. Percent (%) range of Cellularity: Grade 1: < 10%; Grade 2: 11–30%; Grade 3: 31–60%; Grade 4: 61–89%; Grade 5: > 90%).

Bone marrow cellularity and architecture of CD2F1 mice treated 24 h pre-TBI with vehicle or JNJ‑26366821 were evaluated (Fig. 5). Authors reported that following radiation, hematopoietic stem cell (HSC) niche factors, including Stem Cell Factor (SCF) in bone marrow are depleted while adipocytes become populated to fill the empty spaces and secrete SCF to promote HSC recovery after irradiation^31^. In this study, bone marrow cellularity was determined by evaluating the amount of adipose (fat) tissue versus hematopoietic cells (minus, mature red blood cells) on one (10x) high power field (HPF). To score cellularity, a grade was assigned, which correlated with a “percentage range” of cellularity; an average was obtained for each group. The grading scheme was: Grade 1: < 10%; Grade 2: 11–30%; Grade 3: 31–60%; Grade 4: 61–89%; Grade 5: > 90% cellularity (Fig. 5, blue bars on graph). Irradiated saline (Veh) treated groups were compared to the respective non-irradiated controls. Samples were collected on different days post-TBI up to day 30. The extent of recovery from radiation damage was estimated from the H&E-stained slides and quantitated as number of megakaryocytes and % cellularity (Fig. 5). Megakaryocytes were evaluated by averaging the number of cells per 10 (40×) high power fields (HPFs)^27^. When compared to non-irradiated controls, irradiated samples show significant damage in the vehicle treated group in comparison to the JNJ‑26366821 treated group on day 1. On day 3, megakaryocytes counts were significantly lower in both irradiated groups. However, by day 7, the JNJ‑26366821 treated group showed significant recovery. By day 15, there were significant differences in the irradiated vehicle-treated and drug treated groups with respect to number of megakaryocytes as well as % cellularity. By day 30, even though the vehicle treated groups recovered, cellularity remained lower than the drug treated group.

### Attenuation of radiation-induced markers of bone marrow and endothelial damage in CD2F1 male mice

**Figure 6:**
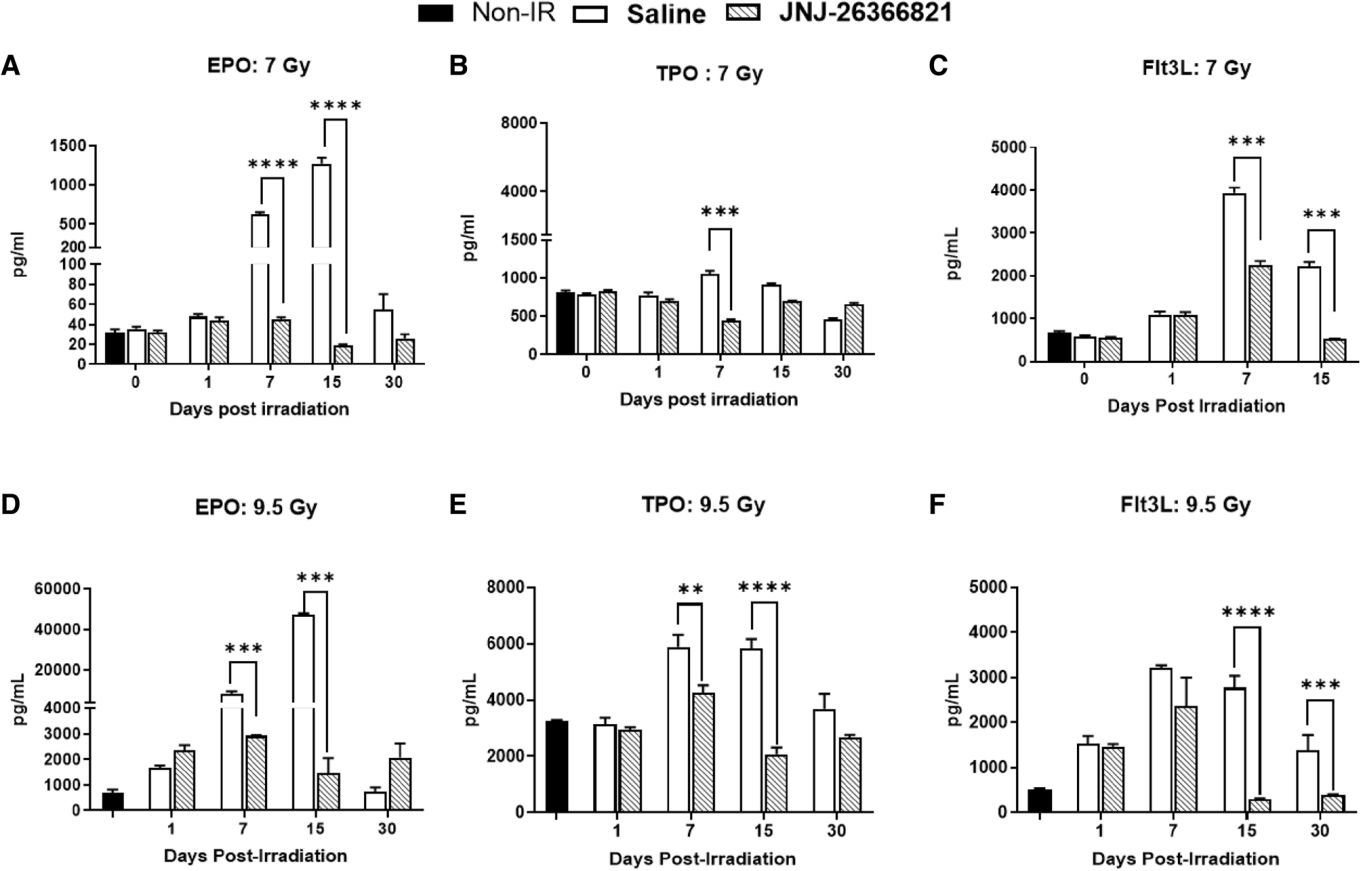
Effect of JNJ‑26366821 (0.3 mg/kg) on circulating markers of bone marrow aplasia in mice exposed to 7 (non-lethal) and 9.5 Gy (lethal) doses. Radiation causes a significant increase in (**A**,**D**) EPO, (**B**,**E**) TPO and (**C**,**F**) FLT3L. At 15 days post-TBI, EPO and TPO levels approached baseline values in JNJ‑26366821-treated animals while they were still severely increased in mice treated with vehicle alone. For FLT3L, baseline reached at day 30 post-TBI. There was dose-dependent increase in EPO (7 vs. 9.5 Gy) and TPO (7 vs. 9.5 Gy) were observed.

EPO, TPO, and Flt3L are known biomarkers of radiation-induced hematopoietic damage. To understand the role of these markers in protecting animals from H-ARS induced lethality mediated by JNJ‑26366821, pre-treatment, serum concentrations were evaluated by ELISA from blood samples collected on days 0 (2 h post-TBI), 1, 7, 14, and 30 post-TBI from animals exposed at 7 and 9.5 Gy (Fig. 6A–F). Serum samples from three groups (non-irradiated control saline treated, irradiated saline and JNJ‑26366821 treated) of mice were compared. The levels in the non-irradiated control group were then compared to the levels observed in 7 and 9.5 Gy irradiated vehicle-treated (saline) and JNJ‑26366821 treated groups.

For animals exposed to 7 Gy (Fig. 6A–C), on days 7 and 15, 13 and 65× higher levels of EPO, respectively, were observed in the irradiated saline group compared to the irradiated JNJ-26366821 group (Fig. 6A, P < 0.0001). On days 7 and 15, statistically higher levels of TPO were observed in the irradiated saline group compared to the irradiated JNJ-26366821 group (Fig. 6B, P < 0.0001). On day 30, statistically lower levels of TPO were observed in the irradiated saline group compared to the irradiated JNJ-26366821 group (Fig. 6B, P < 0.0001). On days 7 and 15, statistically higher levels of FLT3L were observed in the 7 Gy irradiated saline group compared to the irradiated JNJ-26366821 group (Fig. 6C, P < 0.0001).

For animals exposed to 9.5 Gy (Fig. 6D–F), on days 7 and 15, statistically higher levels of EPO were observed in the irradiated saline group compared to the irradiated JNJ-26366821 group (Fig. 6D, P < 0.0092), furthermore. On days 7 and 15, statistically higher levels of TPO were observed in the irradiated saline group compared to the irradiated JNJ-26366821 group (Fig. 6E, P < 0.0078). On days 15 and 30, statistically higher levels of FLT3L were observed in the irradiated saline group compared to the irradiated JNJ-26366821 group (Fig. 6F, P < 0.0001).

**Figure 7:**
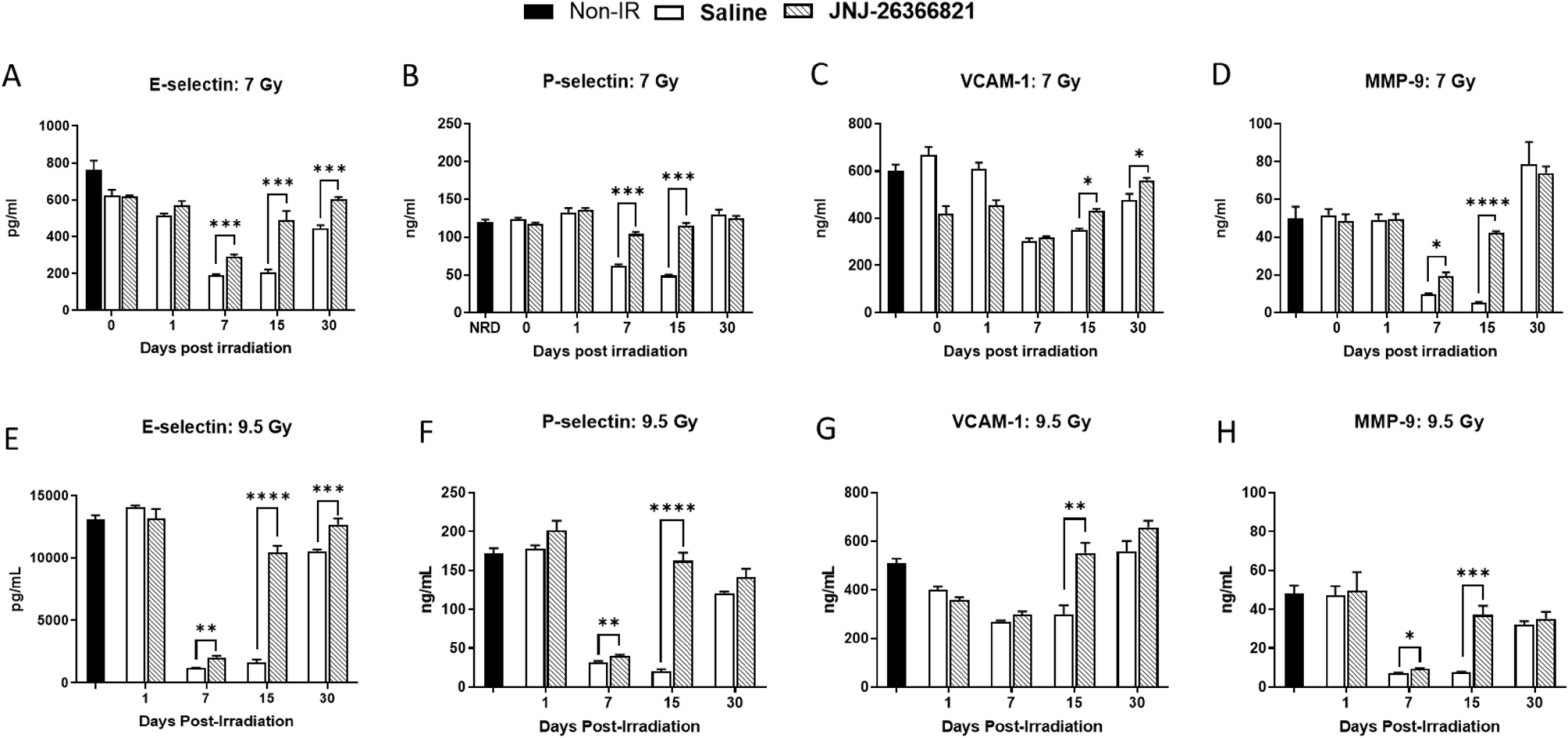
Effect of JNJ‑26366821 (0.3 mg/kg) on circulating markers of endothelial dysfunction in mice exposed to 7 (non-lethal) and 9.5 Gy (lethal) doses. Radiation caused decrease in the in circulating (**A**,**E**) E-selectin, (**B**,**F**) P-selectin, (**C**,**G**) VCAM and (**D**,**H**) MMP9. At 15 days post-TBI, levels approached baseline values in JNJ‑26366821-treated animals while they were still severely reduced in mice treated with vehicle alone. There was dose-dependent decrease in MMP-9 (7 vs. 9.5 Gy) was observed. Data represented are mean ± standard error of the mean (SEM) for n = 6 mice per group.

To evaluate potential mechanisms for the beneficial effects of JNJ‑26366821 on radiation-induced mortality and its effects on endothelial damage, serum concentrations of E-selectin, P-selectin, VCAM, and MMP-9 were measured. Levels of three of the four endothelial membrane proteins, E-selectin, P-selectin, and MMP-9 showed a significant reduction at 7 and 15 days after irradiation at both 7 and 9.5 Gy (Fig. 7A–H). However, pre-administration of JNJ‑26366821 attenuated the inflammatory response and approaching baseline values whereas the levels in vehicle-treated animals were still significantly low. By day 30 post-TBI, serum concentration of E-selectin, P-selectin, and MMP-9 in vehicle treated groups also increased significantly. In the case of VCAM, serum concentration was significantly reduced on day 15 after exposure in the vehicle control group, whereas JNJ‑26366821 pre-treatment alleviated the inflammatory response while approaching the baseline value.

For animals exposed to 7 Gy, on day 7, statistically higher levels of E-selectin were observed in the irradiated JNJ-26366821 group compared to the irradiated saline group (Fig. 7A, P = 0.0001). On days 15 and 30, statistically higher levels of E-Selectin were observed in the irradiated JNJ-26366821 group compared to the irradiated saline group (Fig. 7A, P = 0.0003). On days 7 15 and 30, statistically higher levels of P-selectin were observed in the irradiated JNJ-26366821 group compared to the irradiated saline group (Fig. 7B, P < 0.0001). On days 1 15 and 30, statistically higher levels of VCAM-1 were observed in the irradiated saline group compared to the irradiated JNJ-26366821 group (Fig. 7C, P < 0.002). On days 7 and 15, statistically higher levels of MMP-9 were observed in the irradiated JNJ-26366821 group compared to the irradiated saline group (Fig. 7D, P < 0.006).

For animals exposed to 9.5 Gy, on days 7, 15and 30, statistically higher levels of E-selectin were observed in the irradiated JNJ-26366821 group compared to the irradiated saline group (Fig. 7E, P ≤ 0.001). On days 7 and 15, statistically higher levels of P-selectin were observed in the irradiated JNJ-26366821 group compared to the irradiated saline group (Fig. 7F, P ≤ 0.0065). On day 15, statistically higher levels of VCAM-1 were observed in the irradiated JNJ-26366821 group compared to the irradiated saline group (Fig. 7G, P = 0.0016). On days 7 and 15, statistically higher levels of MMP-9 were observed in the irradiated JNJ-26366821 group compared to the irradiated saline group (Fig. 7H, P ≤ 0.0001).

## Discussion

With increasing nuclear threats around the world, there is a need for additional medical countermeasures to mitigate radiation injury and mortality. In particular, there is a need for radiation countermeasures that can be administered prior to radiation exposure to protect military personnel and first responders before deployment in a radiation-exposed field since the only approved products are for post-TBI exposure^21^. Here we report the first evaluations of JNJ‑26366821 as a radiation prophylactic agent in three mouse strains (CD2F1 males, C3H/HeN males, and C57BL/6J males and females). There are data in multiple animal models evaluating the safety and pharmacology of JNJ-26366821 that have supported its evaluation in human clinical studies^22,32^. In a 14-day acute toxicity study, we also reported a single dose of 3 mg/kg is safe in CD2F1 male mice^29^. Here, our results demonstrate that a single dose of JNJ‑26366821 provides a highly significant survival benefit in lethally irradiated mice, with a window of protection between 2 and 24 h pre-radiation exposure. Pre-administration of JNJ‑26366821 also accelerates peripheral blood cells recovery, stimulates megakaryopoiesis and multi-lineage hematopoietic recovery, attenuates biomarkers of hematopoietic damage in blood, and shows evidence of vascular protection. A significant observation is that all mice, treated with a single dose of JNJ‑26366821, survived a supralethal dose (11 Gy) of radiation. A single dose of JNJ‑26366821 administered either 2 h, 12 h, or 24 h prior to TBI, protected ~ 100% of CD2F1 mice exposed to lethal dose of radiation and was efficacious at all doses (0.1 to 3 mg/kg) tested.

To assess potential mechanisms by which JNJ-26366821 provides such a pronounced survival benefit as a prophylactic countermeasure, it is instructive to look at the factors that drive mortality in H-ARS. In H-ARS, acute whole body radiation exposure induces apoptotic/mitotic death of hematopoietic progenitors, leading to depletion of lympho-hematopoietic elements and immunosuppression, as shown by neutropenia, thrombocytopenia and bone marrow hyperplasia^8,9^. It has also been shown that radiation-induced neutropenia and thrombocytopenia contribute significantly to lethality in the first 30-days post-TBI because of damage to the progenitor cells in the bone marrow and damage to the bone marrow niche, which leads to hemorrhage and bacterial infections. In addition, in published studies, it has been shown that production of hematopoietic cytokines, EPO, TPO, and Flt3L are increased after radiation^27,33,34^ as inflammatory response to radiation exposure. TPO, a hematopoietic growth factor regulates megakaryocyte and platelet production, mainly produced by liver and kidney^35,36^. Previous work highlighted the role of Flt3L as a radiation specific biomarker for the extent of bone marrow damage due to H-ARS^34^. EPO is a hepatic and renal glycoprotein, which regulates erythropoiesis. EPO promotes the proliferation and differentiation of erythroid progenitor cells thereby increasing survival^34^.

In this study JNJ‑26366821 increased neutrophils and platelets in non-lethally (7 Gy) irradiated mice at several time-points during the initial 30-days following irradiation. This sublethal radiation dose induces severe bone marrow myelosuppression in this mouse model^26^. JNJ‑26366821 promotes an increase in circulating platelets, which supports the hypothesis that increased survival as a result of JNJ‑26366821 pre-administration may be in part a result of the thrombopoietic effects of the compound. JNJ‑26366821 interacts with the cMpl receptor, therefore it’s ability to rescue mice from radiation-induced lethality may be a result of accelerated recovery of progenitor cells that stimulate bone marrow production^7,11,35,37^. Among the first identified roles of TPO was in the maturation of megakaryocytes and the fragmentation into platelets^38^. Platelets serve vital roles in wound repair and the innate immune response. In general, TPO levels in the blood are inversely related to platelet counts, likely because the c-Mpl receptor is present on mature platelets meaning more platelets allows absorption of more TPO, less platelets leads to more circulating TPO in the blood^9,11,14^. In the bone marrow, it has been indicated that mRNA expression of TPO is elevated in response to reduced platelet levels^39^. This leads to an increase in blood cell formation and faster recovery of progenitor cells, a role which has also been established for JNJ‑26366821^29^. TPO and its receptor c-Mpl are master regulators of both megakaryopoiesis and HSCs^40,41^. TPO was found to increase the repair of double strand breaks induced by various DNA damaging agents. Romiplostim (Nplate), a thrombopoietin mimetic, was also found to repair both short-term and long-term radiation-induced accumulation of DNA damage in HSCs^40,41^. Thus, our results may have important clinical implications in the use of TPO agonists prior to sending the troops to battle field as well as before radiotherapy to minimize the risk of long-term residual hematopoietic stem and progenitor cells injury.

The data herein also show that there is a gradual increase in serum EPO, TPO, and FLT3L levels in irradiated animals, a compensatory response to bone marrow damage while animals were manifesting H-ARS with a peak 7–15 days post-exposure^27^. JNJ‑26366821 pre-administration significantly reduced the concentration of these biomarkers in serum which can be correlated to the bone marrow regeneration and survival of the animals from lethal dose of ionizing radiation exposure.

Radiation-induced vascular injury also plays a critical role in the mechanism of early to delayed radiation responses in many different organ systems^42^. Endothelial apoptosis is thought to promote acute epithelial injury^42–44^ in the gut which causes development of delayed radiation enteropathy^44^. Endothelial dysfunction markers have also been shown to play a prominent role in pulmonary radiation toxicity^42,45,46^ as delayed response. We have shown alteration of these markers (MMP-9, E-selectin, P-selectin, and VCAM) in JNJ‑26366821 pre-treated mice at early days (7–15 post-exposure) suggesting a potential impact of JNJ-26366821 on vascular endothelial injury and recovery.

Other TPO mimetics have been utilized in conditions where patients have thrombocytopenia and there is a need to elevate platelet counts such as Idiopathic Thrombocytopenia Purpura, Aplastic Anemia, or Chemotherapy-induced thrombocytopenia. Data from this study raise the possibility of using JNJ-26366821 clinical applications where there is a need to treat or prevent radiation induced toxicity such as myeloablative bone marrow transplant.

This manuscript describes our efforts to demonstrate the efficacy of JNJ-26366821 as a promising prophylactic radiation countermeasure. As with all countermeasure development, this is done following the guidance of the FDA animal rule. This work has demonstrated efficacy in various strains of mice. However, in line with the animal rule the compound should also be tested in additional species (e.g. nonhuman primates or another large animal model). In addition, the current work focused on a specific age that equates to young, sexually mature adults. It would be ideal to confirm efficacy at additional age groups such as pediatric and geriatric. We retained the focus of young adults in this study as it more closely mirrors the average age group of current US military personnel. Finally, we demonstrated efficacy in both sexes for C57BL/6J strain, ideally efficacy could be established in both sexes for all strains used.

## Conclusion

In summary, the data in this study support the potential for JNJ-26366821 to be a safe and effective prophylactic radiation countermeasure which has a broad window for medical management from 24 to 2 h prior to TBI. The drug protects the hematopoietic system and preventing lethal radiation injury. A broad window of protection as well as its efficacy in three different strains of mice with a single dose of the drug make JNJ‑26366821 a promising radiation countermeasure for military members or first responders within a diverse population.

### Supplementary Information

Supplementary Figures.

## Data Availability

All data generated or analyzed during this study are included in this published article and its supplementary information files.
