# Supplementary material for: PEGylated thrombopoietin mimetic, JNJ‑26366821 a novel prophylactic radiation countermeasure for acute radiation injury
Source: Sci Rep. 2023 Sep 14;13:15211. doi: 10.1038/s41598-023-42443-0 (PMC10502090; doi:10.1038/s41598-023-42443-0)

Supl Fig 1. Survival of CD2F1 mice following total body irradiation with 9.35 Gy. A single dose of JNJ‑26366821 at 0.3 mg/kg (●) or saline as a vehicle (○) was administered 24 h prior to TBI. Kaplan-Meier survival curves were plotted using GraphPad software; n=24 mice per group and trend in survival is compared between vehicle and drug-treated groups (Log-rank test p = 0.0061).

Supl Fig 2. JNJ‑26366821 (0.3 mg/ kg) accelerates hematopoietic progenitor cell (CFU-GEMM) recovery after a non-lethal dose of radiation (7 Gy). Clonogenic Colony forming units (CFU) assay with femoral bone marrow was carried out on days 0 (2 h post-TBI), 1, 3, 7, 15, and 30 after exposure. Cells from three femurs were pooled, counted, and each sample plated in duplicate to be scored 14 days after plating. Data are expressed as mean ± Standard error of mean (SEM) for individual cell type CFU-GEMM (A), CFU-GM (B) and BFU-E (C). Statistical significance is indicated as *.


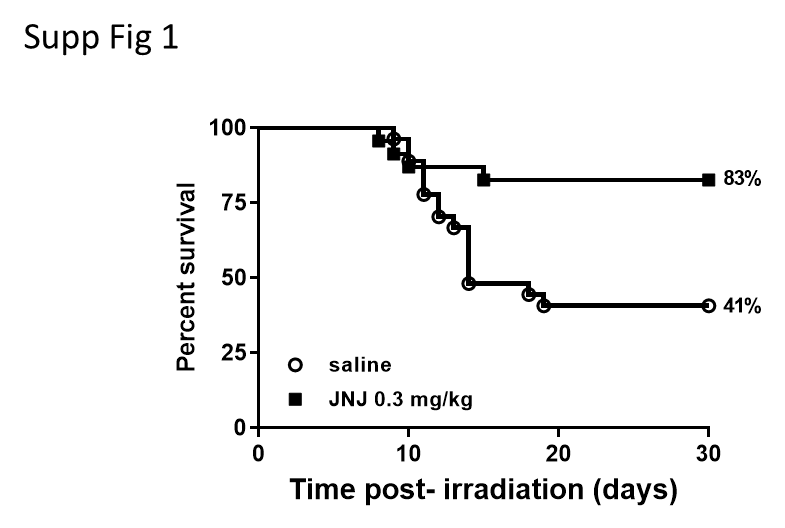


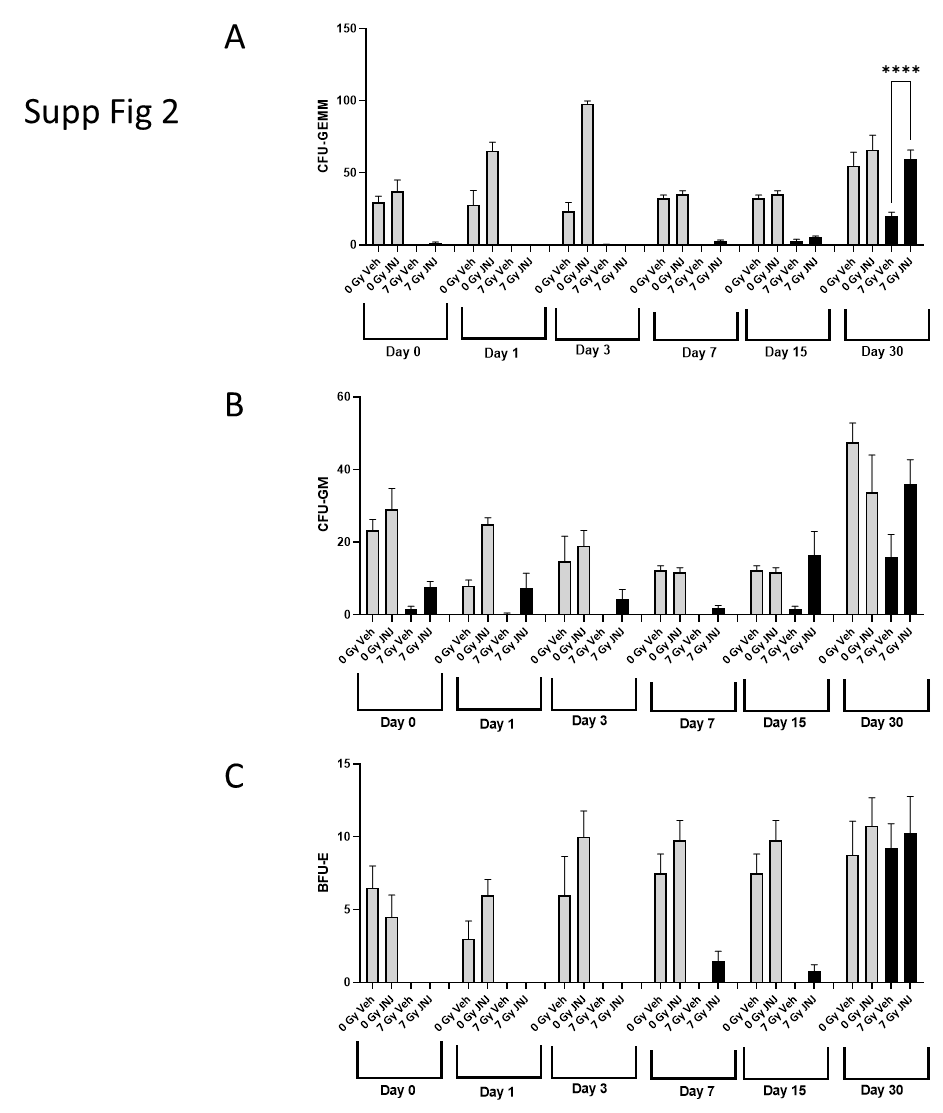

Supplement: Supplementary file 1 — Supplementary Figures. [file 41598_2023_42443_MOESM1_ESM.docx]
